# Trends in the Development of Acupuncture-Related Technologies Based on Patents in South Korea

**DOI:** 10.1155/2024/2204071

**Published:** 2024-04-23

**Authors:** Sung Min Lim, Eunji Go

**Affiliations:** Department of Clinical Research on Rehabilitation, Korea National Rehabilitation Research Institute, Seoul, Republic of Korea

## Abstract

**Background:**

Several studies have assessed the safety and efficacy of acupuncture, but none have reviewed patent trends related to acupuncture as an intervention. This study analyzed trends in patents for the development of acupuncture-related technologies in Korea.

**Methods:**

The Korea Intellectual Property Rights Information Service (KIPRIS) was searched for information on acupuncture-related patents registered until August 11, 2021, without any limit on the search period. Only acupuncture and electro-acupuncture were included in this review. The current status, contents, and technological characteristics of the relevant patents were analyzed systematically to identify the overall status of acupuncture-related patents.

**Results:**

Overall, 126 patents were identified from the KIPRIS database, of which 93 were unrelated to the research topic and were excluded. Finally, 33 patents were assessed. Interest in the development of acupuncture technology has increased steadily over the past decade. Patent applications reached their peak in 2018 with six instances, while patent registration peaked in 2019 with seven instances. The interval between the application and registration of an individual patent was 14.3 months (449 days). Twenty-two acupuncture-related patents were for acupuncture (66.6%) and 11 for electro-acupuncture (33.3%). The technical specifications for the patents included acupuncture devices (30.3%), acupuncture manufacturing methods and equipment (36.4%), and electro-acupuncture devices (33.3%).

**Conclusion:**

Acupuncture-related technology currently focuses on technological advancements for the safe and effective delivery of stimulation. The findings demonstrate trends toward new diverse and complex technological advancements for acupuncture devices, manufacturing methods and equipment, and electro-acupuncture devices.

## 1. Introduction

Acupuncture is the most commonly used form of traditional medicine worldwide. The standard protocol for acupuncture procedures to assure safety and efficacy recommends that the practitioner must select the acupuncture needle after accounting for the physical condition of the patient, disease status, and location of the acupuncture points [1]. Therefore, acupuncture requires consideration of different patient- and device-related characteristics to consolidate therapeutic methods.

Recent studies on the research trends in Korean medicine based on temporal and network analyses and cluster network analysis of keywords reported that the frequency of appearance of “acupuncture” was substantially higher than that of other keywords. It was also found to be the most important method, which always ranked the highest, according to frequency analysis conducted at various time intervals [2].

Numerous clinical trials have been conducted worldwide to assess the efficacy of acupuncture therapy. The STRICTA recommendations include stipulations for the inclusion of the specific type of acupuncture, type of acupuncture stimulation, and insertion depth while reporting the results of interventional clinical trials using acupuncture [3].

The collection and analysis of patent information are employed actively in conjunction with recent advances in scientific and medical technologies to identify the latest trends and innovations in the relevant technologies [4]. Although several studies have reviewed the safety and efficacy of acupuncture, no study has conducted a review of the patent trends related to acupuncture as an intervention. Analyzing patent trends in acupuncture can help in the development of acupuncture-related technologies by incorporating information on current advances. This approach can facilitate the novelty and innovation of acupuncture techniques.

Therefore, the present study aimed to provide an overview of the trends in acupuncture-related patents that have been registered to date. To this end, the study searched and analyzed Korean patents to conduct a systematic analysis of the current status, content, and technological characteristics of relevant patents to establish basic data for developing new acupuncture technologies in the future.

## 2. Materials and Methods

Data for this study were collected by exploring the Korea Intellectual Property Rights Information Service (KIPRIS, https://www.kipris.or.kr/), a search service of patent-related information provided by the Korean Intellectual Property Office (KIPO) that has established a database of all intellectual property (IP)-related information in South Korea. To obtain a patent for a technology related to acupuncture, the first step is to file a patent application with the KIPO. The patent examiners at KIPO will then assess the novelty and inventiveness of the application by reviewing existing patents published both domestically and internationally, before making a decision on whether to grant a patent registration.

The patent database was searched using the term “acupuncture” to identify patents registered as of August 11, 2021, without any limit on the search period. Only acupuncture and electroacupuncture were included in the current analysis, while pharmacopuncture, auricular acupuncture, and cutaneous acupuncture were excluded.

All extracted patents were screened independently and selected by two reviewers. During the preliminary screening, the title and abstract of the searched patents were reviewed based on the inclusion and exclusion criteria to eliminate patents that were determined to be unrelated to the research topic. During the second screening, the full texts of patents with unclear abstracts were reviewed to select those within the purview of the research topic. Any disagreement between the two reviewers was resolved by consensual discussion. Data extraction from the ultimately selected patents was conducted according to the predefined data extraction format.

Sub-analyses were performed for the following parameters to identify the overall status of the acupuncture-related patents: (1) patent application year, registration year, and duration between patent application and registration; (2) patent applicant and inventor; (3) analysis according to the International Patent Classification (IPC); and (4) patent categorization based on a specific technology.

## 3. Results

### 3.1. Search Results

**Figure 1:**
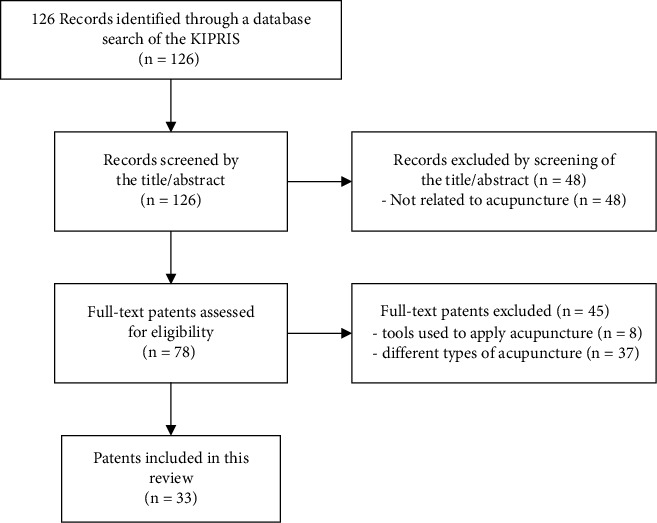
Flow chart of the patent selection process.

The search yielded a total of 126 patents, of which 33 were selected for inclusion in the final analysis. Ninety-three patents that were deemed to be unrelated to the research topic based on the eligibility criteria during the preliminary screening were excluded [patents unrelated to acupuncture (*n* = 48)] and second screening [different types of acupuncture (*n* = 37) and devices used to apply acupuncture (*n* = 8)] (Figure 1). In the preliminary screening, patents related to acupuncture points and acupuncture assistants were excluded as they were not relevant to the purpose of this study.

### 3.2. Patent Application Year, Registration Year, and Duration between Patent Application and Registration

**Figure 2:**
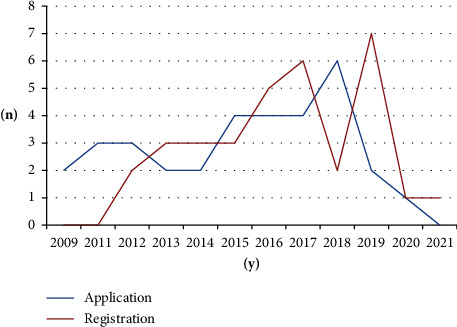
Analysis of patent application and registration trends.

The frequency of patent applications by year was as follows: 2 cases in 2009, which increased gradually to 3 cases each in 2011 and 2012; 4 cases each in 2015, 2016, and 2017; and 6 cases in 2018. However, the number of patent applications subsequently decreased to 2 in 2019 and 1 in 2020. The number of patent registrations included 2 cases in 2012, which increased to 3 each in 2013, 2014, and 2015; 5 in 2016; 6 in 2017; and 7 in 2019. However, the number of patent registrations subsequently decreased to 1 case each in 2020 and 2021 (Figure 2). The mean duration between patent application and registration for an individual patent was 14.3 months (449 days).

### 3.3. Patent Applicants and Inventors

**Figure 3:**
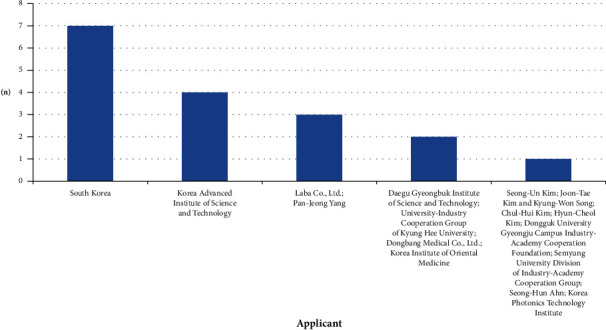
Frequency analysis of applicants.

**Figure 4:**
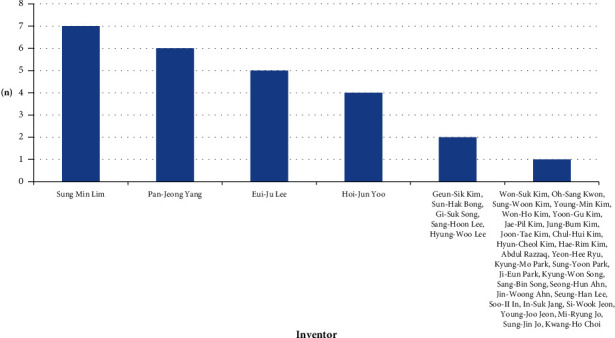
Frequency analysis of inventors.

The National Rehabilitation Center (*n* = 7) held the highest number of patent applications, followed by the Korea Advanced Institute of Science and Technology (*n* = 4), Laba Co. Ltd. (*n* = 3), and Pan-Jeong Yang (*n* = 3) (Figure 3), respectively. The inventor with the highest number of patent applications was Sung-Min Lim (*n* = 7), followed Pan-Jeong Yang (*n* = 6), Eui-Ju Lee (*n* = 5), and Hoi-Jun Yoo (*n* = 4) (Figure 4), respectively.

### 3.4. Analysis According to the IPC

A total of 117 IPC classes that included all IPC classes listed in the 33 patents selected were included in the IPC-based analysis.

IPC class A61 (medical or veterinary science; hygiene) accounted for the highest proportion of patents (*n* = 83, 70.9%). A61H (physical therapy apparatus, e.g., devices for locating or stimulating reflex points on the body; artificial respiration; massage; bathing devices for special therapeutic or hygienic purposes or specific parts of the body) accounted for the highest proportion among IPC class A61 (*n* = 42, 35.8%), followed by A61N (electrotherapy; magnetotherapy; radiation therapy; ultrasound therapy) (*n* = 22, 18.8%) and A61B (diagnosis; surgery; identification) (*n* = 10, 8.5%), respectively. The A61H 39/08 subclass (devices for applying needles to such points, e.g., for acupuncture) was the most common (*n* = 27, 23.1%), followed by A61H 39/00 (devices for locating or stimulating specific reflex points on the body for physical therapy, e.g., acupuncture; *n* = 10, 8.5%) (Supplementary Table 1).

### 3.5. Analysis of Patents with respect to a Specific Technology

**Figure 5:**
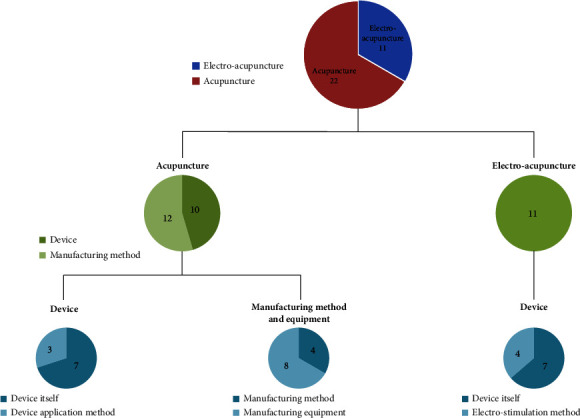
Analysis of specific types of technology for acupuncture-related patents.

Twenty-two of the 33 acupuncture-related patents were for acupuncture (66.6%), and 11 were for electroacupuncture (33.3%). The specific technologies for the patents included acupuncture devices (*n* = 10, 30.3%), acupuncture manufacturing methods and equipment (*n* = 12, 36.4%), and electro-acupuncture devices (*n* = 11, 33.3%) (Figure 5).

#### 3.5.1. Acupuncture Devices

Ten patents were granted for acupuncture devices, including seven patents for the device itself and three patents for the device application method (Table 1).

Patents for acupuncture devices focused on the safety and convenience of the acupuncture procedure, including patents for maintaining the stability of the inserted needles, consolidating the safety of the procedure [5, 6] and increasing the degree of stimulation and convenience of use with multiple needles [7]. Moreover, other patents focused on performing the acupuncture procedure or providing additional stimulation during acupuncture, including patents for an apparatus that could deliver vibrations to the inserted needle [8, 9] and procedures for inducing the therapeutic effect of acupuncture and cupping therapy [10]. Furthermore, another patent for an acupuncture set for double-blinding (required in clinical trials investigating the therapeutic effect) was used in a study for establishing the clinical evidence of the efficacy of acupuncture [11].

Moreover, other patents included assistive techniques for simplifying the acupuncture procedure from the operator's perspective, including an ultrasound-guided technique for accurate intramuscular needle insertion [12]; an endoscopy-guided technique for inserting the acupuncture needle into the nose [13]; and a robotic system for performing acupuncture treatment, which was developed to improve the convenience, accuracy, and effectiveness of the procedure [14].

#### 3.5.2. Acupuncture Manufacturing Methods and Equipment

We found 12 patents on acupuncture manufacturing methods and equipment, including four on manufacturing methods and eight on manufacturing equipment (Table 1).

Patents for manufacturing methods included techniques for creating micro- or nano-sized holes on the surface of the acupuncture needle to maximize the surface area of the working end and facilitate greater stimulation to the acupuncture site [15]; acupuncture needle assembly by combining the needle body and container [16, 17]; and manufacturing and packing processes that coupled the acupuncture needle with the needle bottle [18].

Patents for manufacturing equipment included polishers for making the needle tip sharper and more uniform [19]; equipment for improving productivity and workability by an automatic assembly of the needle body and needle bottle [20–24]; equipment for mass production of acupuncture needles by simplifying and perfecting the series of processes involved in fabricating the finished product (from polishing to assembly) [25]; and intraoral acupuncture production equipment that uses the patient's anthropometric information to align the center of mass of the body with the center of gravity [26].

#### 3.5.3. Electroacupuncture Devices

There were 11 patents on electroacupuncture devices, including seven on devices and four on electrostimulation devices (Table 1).

Patents for electroacupuncture devices included devices that can provide an arbitrary electrical stimulation effect by initiating or controlling a current flow path between the electric needles and reduce electrical resistance compared to conventional electro-acupuncture [27] and electroacupuncture vibrating needles to relieve pain and provide comfort during acupuncture [28]. Patents were also granted on the safety of electroacupuncture, including electroacupuncture with an insulating plate constructed as a single unit to provide insulation to the skin [29] and electroacupuncture that firmly maintains the position of the needle after acupuncture [30]. Other patents included those for electroacupuncture capable of providing electrical stimulation in close proximity to local points that could be used for high-potential or long-term stimulation [31]; apparatus in which the electrical stimulation self-adjusts in response to a sensor that measures bio-signals [32]; and a portable electroacupuncture needle device with a laser irradiating the meridian points corresponding to the patient's disease [33].

Patents for electrostimulation methods included techniques for supplying or controlling electricity to the electroacupuncture needles, including methods that enabled communication between electric needles through a human body medium to eliminate the need to control all needles individually and eradicate instability when connected by a wire [34] and a method for supplying electrostimulation by generating a current through photoelectric conversion without an external power supply [35]. Moreover, there were other patents for an electroacupuncture system that apply the appropriate current to electrical needles based on disease information determined by the detection of the patient's bio and brain wave signals [36] and an electroacupuncture platform that enables electrostimulation by a single point or various channels [37].

## 4. Discussion

The present study aimed to identify the latest trends in the development of acupuncture-related technologies by collecting and analyzing acupuncture-related patent information to present basic data essential for future technological innovation. Therefore, the current status, contents, and technological characteristics of patents registered to date were investigated and analyzed systematically.

Acupuncture is among the most commonly used treatment methods in the world. It is characterized by the insertion of needles into the body for remedial purposes [38, 39]. Essentially, electroacupuncture is a traditional treatment method that applies electrostimulation to specific meridian points to produce potent effects in various neurological diseases [40, 41]. This method can deliver continuous electrostimulation to a part of the body and objectively adjust the amount of stimulation to reduce the stimulation-related adverse effects in localized areas [42].

Interest in the development of acupuncture technology has grown steadily over the past decade. Patent application peaked in 2018 with six cases, while patent registration reached its peak in 2019 with seven cases.

The number of patents for the development of technologies for acupuncture was higher than that for electroacupuncture. The number of patents for manufacturing methods and the development/improvement of manufacturing equipment for acupuncture was slightly higher than that for acupuncture devices. The development of acupuncture devices focused primarily on improving operator convenience during the procedure and providing a combined stimulation effect. These patents included development using modern medical devices, such as ultrasound, endoscopy, and robotics.

Patents for acupuncture manufacturing methods and equipment included improvement of structural coupling for manufacturing and development of an automatic assembly for mass production, as well as manufacturing methods for enhancing acupuncture stimulation through improvements in the needle tip.

Patents for the development of electroacupuncture technologies focused mainly on the devices. The primary objective of these patents was to ensure safety during electroacupuncture stimulation, provide stable electroacupuncture stimulation by improving the electric current supply method, and control electrostimulation via linkage to bio-signal information.

Our findings confirmed that the development of acupuncture-related technologies focused on technical advancement for effective and safe delivery of stimulation. Moreover, the findings also confirmed that the latest research trends pivoted toward diverse and complex technological aspects, including acupuncture devices, manufacturing methods and equipment, and electroacupuncture devices.

These findings are significant since they evinced that technological specialization and overall tendency should be considered simultaneously for ensuring novel and innovative patents for the development of new acupuncture-related technologies. Moreover, by identifying all patents registered to date, the study provided as much information as possible on the current status, contents, and technological characteristics of acupuncture-related technologies. While the goal of the development of acupuncture-related technologies was to enhance the efficacy of acupuncture, recent developmental efforts have been focused on objectively verifying the effectiveness of acupuncture.

This analysis of acupuncture-related patent information reveals the importance of considering in-house development of acupuncture or electroacupuncture devices, attempts to devise systems using modern technology, and delivery of combined stimulation. Moreover, it is possible to discover new applications of vacant technologies for manufacturing special forms of acupuncture equipment or automation. Furthermore, the validities of novel acupuncture technologies must be tested to ensure clinical significance, creating new challenges for the future development of acupuncture-related technologies.

The limitations of the present study include the fact that patents registered outside of South Korea were not included in the analysis due to difficulty in accessing patent-related information. Therefore, future studies should search and analyze acupuncture-related patents registered outside South Korea to investigate more diverse acupuncture-related technological and development trends. It would also be meaningful to conduct clinical trials incorporating previously developed technologies that boost the efficacy of acupuncture as well as recently developed novel technologies for objectively verifying the efficacy and safety of acupuncture.

## 5. Conclusion

The present study systematically analyzed the current status, contents, and technological characteristics of acupuncture-related patents registered in South Korea. The findings confirmed that the development of acupuncture-related technologies focused on technical advancement for effective and safe delivery of stimulation. Moreover, the findings also confirmed the paradigm shift toward diverse and complex technologies for acupuncture devices, manufacturing methods and equipment, and electroacupuncture devices. Hence, the future development of new acupuncture-related technologies requires an approach that considers technological specialization and the overall tendency to ensure the novelty and innovation of patents.

## Figures and Tables

**Table 1:** Status and impact of patents.

Patent number	Application (y)	Registration in other countries	Forward citation
KR101661398 [5]	2014	NA	8
KR102253077 [6]	2020	NA	2
KR102037773 [7]	2019	NA	0
KR101966638 [8]	2018	NA	0
KR101966637 [9]	2018	NA	0
KR101721403 [10]	2016	NA	0
KR102137777 [11]	2018	NA	3
KR101800939 [12]	2015	NA	4
KR101542308 [13]	2013	NA	1
KR101179293 [14]	2009	NA	2
KR101458486 [15]	2014	US, EP, JP, CN	2
KR101756167 [16]	2015	NA	0
KR101675716 [17]	2016	NA	0
KR102060011 [18]	2019	US	4
KR101656867 [19]	2016	NA	6
KR101589516 [20]	2015	NA	2
KR101357293 [21]	2012	NA	6
KR101941240 [22]	2018	NA	0
KR102025183 [23]	2018	NA	0
KR101618206 [24]	2015	NA	3
KR102058023 [25]	2018	NA	2
KR101562056 [26]	2012	NA	0
KR101307775 [27]	2011	NA	9
KR101559212 [28]	2013	NA	1
KR101405060 [29]	2012	NA	3
KR101885469 [30]	2017	NA	1
KR101238727 [31]	2011	NA	1
KR101866758 [32]	2017	NA	0
KR101763561 [33]	2016	NA	2
KR101201296 [34]	2009	NA	5
KR101800997 [35]	2017	NA	1
KR101761724 [36]	2017	NA	1
KR101242553 [37]	2011	NA	2

## Data Availability

All data generated or analyzed during this study are included in this published article.

## Abbreviations

IPC: International patent classification.

## Supplementary Materials

Supplementary Materials Supplementary Table 1: frequency analysis according to the International Patent Classification.
