# Supplementary material for: Trends in the Development of Acupuncture-Related Technologies Based on Patents in South Korea
Source: Evid Based Complement Alternat Med. 2024 Apr 23;2024:2204071. doi: 10.1155/2024/2204071 (PMC11074822; doi:10.1155/2024/2204071)
Supplement: Supplementary Materials — Supplementary Table 1: frequency analysis according to the International Patent Classification. [file 2204071.f1.pdf]

Supplementary Table 1. Frequency analysis according to the International Patent Classification

Class		No.	%	Explanation
**A61 (MEDICAL OR VETERINARY SCIENCE; HYGIENE)**	**B (DIAGNOSIS; SURGERY; IDENTIFICATION)**			
	001/233	1	0.9	- for the nose, i.e., nasoscopes
	005/00	3	2.6	Measuring for diagnostic purposes; Identification of persons
	005/0402	1	0.9	Electrocardiography, i.e., ECG
	005/0476	1	0.9	Electroencephalography
	005/053	1	0.9	Measuring electrical impedance or conductance of a portion of the body
	005/103	1	0.9	Measuring devices for testing the shape, pattern, size, or movement of the body or parts thereof for diagnostic purposes
	008/00	1	0.9	Diagnosis using ultrasonic, sonic, or infrasonic waves
	008/08	1	0.9	Detecting organic movements or changes, e.g., tumors, cysts, swellings
	**C (DENTISTRY; ORAL OR DENTAL HYGIENE)**			
	007/08	1	0.9	Mouthpiece-type retainers
	013/34	1	0.9	Making or working of models, e.g., preliminary castings, trial dentures; Dowel pins
	**F (FILTERS IMPLANTABLE INTO BLOOD VESSELS; PROSTHESES; DEVICES PROVIDING**			

	**PATENCY TO, OR PREVENTING COLLAPSING OF, TUBULAR STRUCTURES OF THE BODY, e.g., STENTS; ORTHOPAEDIC, NURSING, OR CONTRACEPTIVE DEVICES; FOMENTATION; TREATMENT OR PROTECTION OF EYES OR EARS; BANDAGES, DRESSINGS OR ABSORBENT PADS; FIRST-AID KITS)**			
	005/00	1	0.9	Orthopedic methods or devices for non-surgical treatment of bones or joints; Nursing devices
	007/00	1	0.9	Heating or cooling appliances for medical or therapeutic treatment of the human body
	013/00	2	1.7	Bandages or dressings; Absorbent pads
	013/02	1	0.9	Adhesive plasters or dressings
	**H<br>(PHYSICAL THERAPY APPARATUS, e.g., DEVICES FOR LOCATING OR STIMULATING REFLEX POINTS IN THE BODY; ARTIFICIAL RESPIRATION; MASSAGE; BATHING DEVICES FOR SPECIAL THERAPEUTIC OR HYGIENIC PURPOSES OR SPECIFIC PARTS OF THE BODY)**			
	009/00	1	0.9	Pneumatic or hydraulic massage
	023/02	1	0.9	- with electric or magnetic drive
	039/00	10	8.5	Devices for locating or stimulating specific reflex points of the body for physical therapy, e.g., acupuncture
	039/02	3	2.6	Devices for locating such points
	039/08	27	23.1	Devices for applying needles to such points, i.e., for acupuncture
	**M<br>(DEVICES FOR INTRODUCING MEDIA INTO, OR ONTO, THE BODY; DEVICES FOR**			

	**TRANSDUCING BODY MEDIA OR FOR TAKING MEDIA FROM THE BODY; DEVICES FOR PRODUCING OR ENDING SLEEP OR STUPOR)**			
	021/00	1	0.9	Other devices or methods to cause a change in the state of consciousness; Devices for producing or ending sleep by mechanical, optical, or acoustical means, e.g., for hypnosis
	037/00	1	0.9	Other apparatus for introducing media into the body; Percutany, i.e., introducing medicines into the body by diffusion through the skin
	**N (ELECTROTHERAPY; MAGNETOTHERAPY; RADIATION THERAPY; ULTRASOUND THERAPY)**			
	001/04	2	1.7	Electrodes
	001/05	6	5.1	- for implantation or insertion into the body, e.g., heart electrode
	001/18	2	1.7	Applying electric currents by contact electrodes
	001/32	1	0.9	- alternating or intermittent currents
	001/36	9	7.7	- for stimulation, e.g. heart pace-makers
	005/06	2	1.7	- using light
**B21 (MECHANICAL METAL-WORKING WITHOUT ESSENTIALLY REMOVING**	**C (MANUFACTURE OF METAL SHEETS, WIRE, RODS, TUBES, OR PROFILES, OTHERWISE THAN BY ROLLING; AUXILIARY OPERATIONS USED IN CONNECTION WITH METAL-WORKING WITHOUT ESSENTIALLY REMOVING MATERIAL)**			
	001/00	1	0.9	Manufacture of metal sheets, metal wire, metal rods, metal tubes by drawing
	**F (WORKING OR PROCESSING OF METAL WIRE)**			

**MATERIAL; PUNCHING METAL)**	003/02	1	0.9	- helically
	045/00	1	0.9	Wire-working in the manufacture of other particular articles
	**G (MAKING NEEDLES, PINS, OR NAILS OF METAL)**			
	001/00	2	1.7	Making needles used for performing operations
	001/08	1	0.9	- of hollow needles or needles with hollow ends, e.g., hypodermic needles, larding-needles
	001/12	2	1.7	Securing, cleaning-off burrs, reconditioning, polishing, grinding
**B23 (MACHINE TOOLS; METAL-WORKING NOT OTHERWISE PROVIDED FOR)**	**K (SOLDERING OR UNSOLDERING; WELDING; CLADDING OR PLATING BY SOLDERING OR WELDING; CUTTING BY APPLYING HEAT LOCALLY, e.g., FLAME CUTTING; WORKING BY LASER BEAM)**			
	026/03	1	0.9	Observing, e.g., monitoring, the workpiece
	026/06	1	0.9	Shaping the laser beam, e.g., by masks or multi-focusing
	026/21	1	0.9	- by welding
	026/324	1	0.9	- involving non-metallic parts
	026/70	1	0.9	Auxiliary operations or equipment
	037/04	1	0.9	- for holding or positioning work
	**P (METAL-WORKING NOT OTHERWISE PROVIDED FOR; COMBINED OPERATIONS; UNIVERSAL MACHINE TOOLS)**			
	015/00	2	1.7	Making specific metal objects by operations not covered by a single other

				subclass or a group in this subclass
	019/10	2	1.7	Aligning parts to be fitted together
	023/04	3	2.6	- for both machining and other metal-working operations
**B24 (GRINDING; POLISHING)**	**B (MACHINES, DEVICES, OR PROCESSES FOR GRINDING OR POLISHING; DRESSING OR CONDITIONING OF ABRADING SURFACES; FEEDING OF GRINDING, POLISHING, OR LAPPING AGENTS)**			
	019/16	1	0.9	- for grinding sharp-pointed workpieces, e.g., needles, pens, fish hooks, tweezers or record player styli
	029/08	1	0.9	- the cross-section being circular, e.g., tubes, wires, needles
	041/02	1	0.9	Frames; Beds; Carriages
**B25 (HAND TOOLS; PORTABLE POWER-DRIVEN TOOLS; MANIPULATORS)**	**J (MANIPULATORS; CHAMBERS PROVIDED WITH MANIPULATION DEVICES)**			
	009/00	1	0.9	Program-controlled manipulators
**B29 (WORKING OF PLASTICS; WORKING OF SUBSTANCES IN A PLASTIC**	**C (SHAPING OR JOINING OF PLASTICS; SHAPING OF MATERIAL IN A PLASTIC STATE, NOT OTHERWISE PROVIDED FOR; AFTER-TREATMENT OF THE SHAPED PRODUCTS, e.g., REPAIRING)**			
	059/02	1	0.9	- by mechanical means, e.g., pressing

**STATE IN GENERAL)**				
**B65 (CONVEYING; PACKING; STORING; HANDLING THIN OR FILAMENTARY MATERIAL)**	**B (MACHINES, APPARATUS, OR DEVICES FOR, OR METHODS OF, PACKAGING ARTICLES OR MATERIALS; UNPACKING)**			
	015/00	1	0.9	Attaching articles to cards, sheets, strings, webs, or other carriers
	**D (CONTAINERS FOR STORAGE OR TRANSPORT OF ARTICLES OR MATERIALS, e.g., BAGS, BARRELS, BOTTLES, BOXES, CANS, CARTONS, CRATES, DRUMS, JARS, TANKS, HOPPERS, FORWARDING CONTAINERS; ACCESSORIES, CLOSURES, OR FITTINGS THEREFOR; PACKAGING ELEMENTS; PACKAGES)**			
	085/24	1	0.9	- for needles, nails, or similar small elongated articles
**C09 (DYES; PAINTS; POLISHES; NATURAL RESINS; ADHESIVES; COMPOSITIONS NOT OTHERWISE PROVIDED FOR; APPLICATIONS OF MATERIALS**	**J (ADHESIVES; NON-MECHANICAL ASPECTS OF ADHESIVE PROCESSES IN GENERAL; ADHESIVE PROCESSES NOT PROVIDED FOR ELSEWHERE; USE OF MATERIALS AS ADHESIVES)**			
	133/08	1	0.9	Homopolymers or copolymers of acrylic acid esters
	171/02	1	0.9	Polyalkylene oxides
	175/04	1	0.9	Polyurethanes
	189/00	1	0.9	Adhesives based on proteins; Adhesives based on derivatives thereof

**NOT OTHERWISE PROVIDED FOR**				
**C25 (ELECTROLYTIC OR ELECTROPHORETIC PROCESSES; APPARATUS THEREOF)**	**B (ELECTROLYTIC OR ELECTROPHORETIC PROCESSES FOR THE PRODUCTION OF COMPOUNDS OR NON-METALS; APPARATUS THEREOF**			
	001/04	1	0.9	- by electrolysis of water
**G02 (OPTICS)**	**B (OPTICAL ELEMENTS, SYSTEMS, OR APPARATUS)**			
	027/20	1	0.9	- for imaging minute objects, e.g., light-pointer
**Total**		117	100	
